# Supplementary material for: Single-Cell Imaging Reveals That Staphylococcus aureus Is Highly Competitive Against Pseudomonas aeruginosa on Surfaces
Source: Front Cell Infect Microbiol. 2021 Aug 26;11:733991. doi: 10.3389/fcimb.2021.733991 (PMC8426923; doi:10.3389/fcimb.2021.733991)

Supplementary Material

Single-cell imaging reveals that *Staphylococcus aureus* is highly competitive against *Pseudomonas aeruginosa* on surfaces

Running Title: Bacterial single-cell interactions on surfaces

Selina Niggli, Tobias Wechsler, Rolf Kümmerli

This file contains the following supplementary materials:

- 2 Supplementary tables
- 5 Supplementary figures
- 4 Supplementary analysis panels that describe the data shown in the corresponding supplementary movie

Supplementary Tables

Supplementary Table S1. Strains used for this study.

**Species and strain name**	**Origin**	**Description**	**Fluorophore**	**Reference**
***Pseudomonas aeruginosa* (PA)**				
PAO1	Wound	Commonly used *P. aeruginosa* laboratory strain.	(1) *ptac::gfp*<br>(2) *Promoterless::mcherry-Promoterless::gfp*<br>(3) *lasR::mcherry-rpsL::gfp*<br>(4) *rhlR::mcherry-rpsL::gfp*	ATCC 15692
***Staphylococcus aureus* (SA)**				
Cowan I	Septic arthritis	MSSA isolate. Highly invasive, but not cytotoxic. Agr-defective.	untagged	ATCC 12598
6850	Osteomyelitis	MSSA isolate. Highly invasive, cytotoxic, and hemolytic.	untagged	ATCC 53657
JE2	Skin and soft tissue infection	USA300 CA-MRSA isolate. Highly virulent, cytotoxic, and hemolytic.	untagged	NARSA

All fluorescent constructs in PAO1 were chromosomally inserted using the mini-Tn7 insertion system.
CA-MRSA: Community-acquired methicillin-resistant *S. aureus*
MSSA: Methicillin-sensitive *S. aureus*
Agr: Accessory gene regulator

Supplementary Table S2. Descriptive statistics of the gene expression analysis, showing the mean expression level ± standard error (SE), the coefficient of variation (cv) and the number of PA cells imaged (N), split according to (i) genes, (ii) strain combinations, and (iii) timepoints. Note that the *rpsL* expression values are merged for two double reporter constructs (PAO1*lasR::mcherry-rpsL::gfp* and PAO1*rhlR::mcherry-rpsL::gfp*). Data is from three independent experiments.

		**Timepoint 1 (5 hours)**			**Timepoint 2 (8 hours)**		
		lasR	***rhlR***	***rpsL***	***lasR***	***rhlR***	***rpsL***
**PA alone**	**mean ± SE**	-0.067 ± 0.004	0.530 ± 0.005	1.685 ± 0.004	0.227 ± 0.003	0.193 ± 0.004	1.069 ± 0.002
	**cv**	-3.629	0.706	0.215	1.009	1.554	0.194
	**N**	4088	6113	10201	7505	5768	13273
**PA + Cowan I**	**mean ± SE**	0.586 ± 0.006	0.962 ± 0.005	2.403 ± 0.006	0.084 ± 0.003	0.081 ± 0.008	1.183 ± 0.002
	**cv**	0.500	0.312	0.202	2.017	6.667	0.156
	**N**	2759	3972	6731	4402	4425	8827
**PA + 6850**	**mean ± SE**	0.306 ± 0.005	0.590 ± 0.011	2.044 ± 0.006	0.241 ± 0.004	0.407 ± 0.005	1.160 ± 0.002
	**cv**	0.782	0.931	0.212	0.981	0.748	0.164
	**N**	2245	2602	4847	4284	3379	7663
**PA + JE2**	**mean ± SE**	0.417 ± 0.007	0.868 ± 0.009	1.882 ± 0.005	0.103 ± 0.003	0.369 ± 0.005	1.121 ± 0.002
	**cv**	0.984	0.597	0.233	1.828	0.745	0.136
	**N**	3509	3066	6575	3386	3016	6402

Supplementary Figures

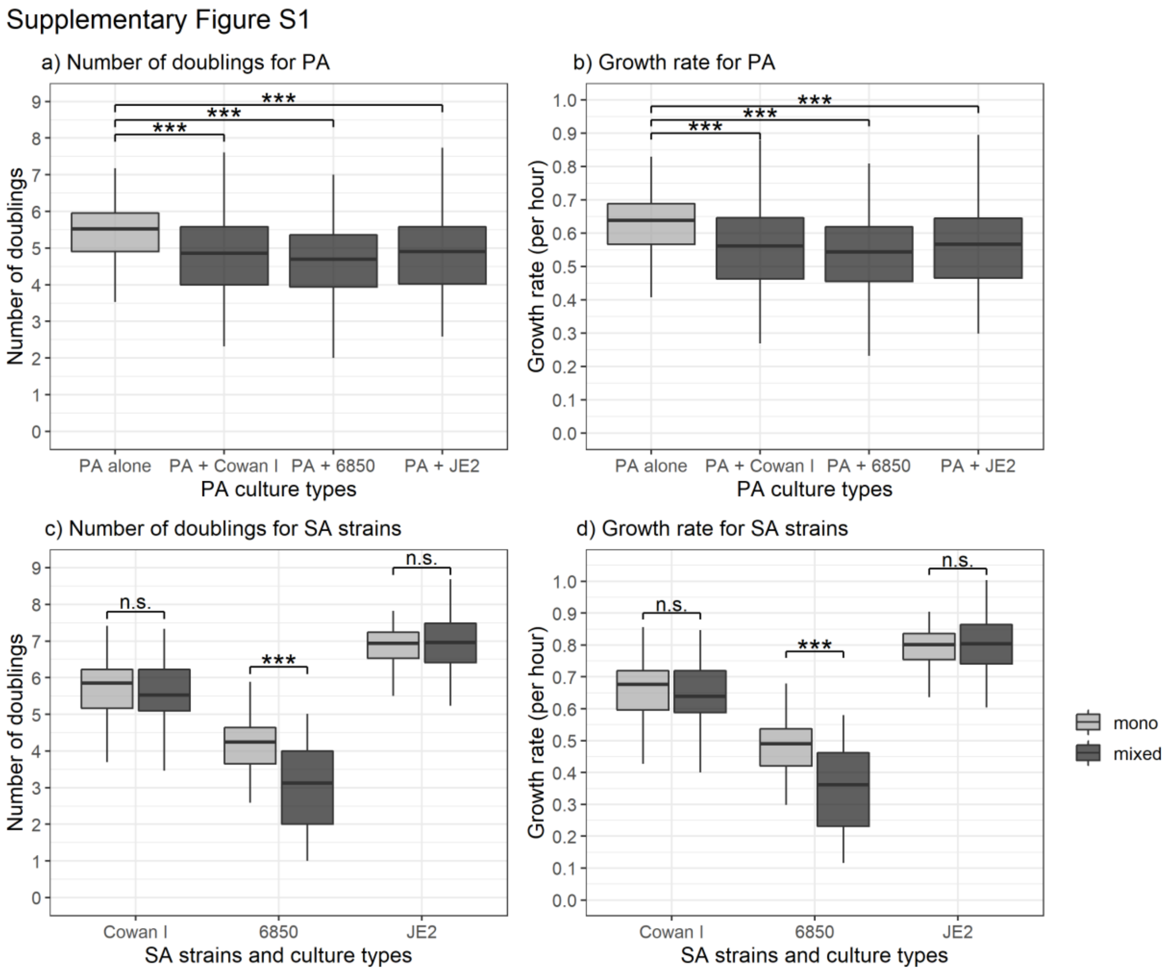

Supplementary Figure S1. Number of doublings and growth rate for *P. aeruginosa* (PA) and *S. aureus* (SA) microcolonies growing in mono- (light-grey) and mixed culture (dark-grey). a) PA performs significantly less doublings in the presence of all three SA strains. b) Growth rate of PA is reduced in the presence of all three SA strains. c) Whereas the number of doublings in Cowan I and JE2 is not affected by the presence of PA, 6850 performs significantly less doublings in mixed microcolonies together with PA. d) Growth rate of Cowan I and JE2 is unaffected by the presence of PA, while 6850 suffers from a reduced growth rate. The box plots show the median (bold line) with the first and the third quartiles. The whiskers cover the 1.5* inter-quartile range (IQR) or extend from the lowest to the highest value if they fall within the 1.5* IQR. *** $p < 0.001$, n.s., not significant. Data is from three independent experiments per PA-SA combination, with a total of 352 and 323 microcolonies for PA and SA strains, respectively.

Supplementary Figure S2

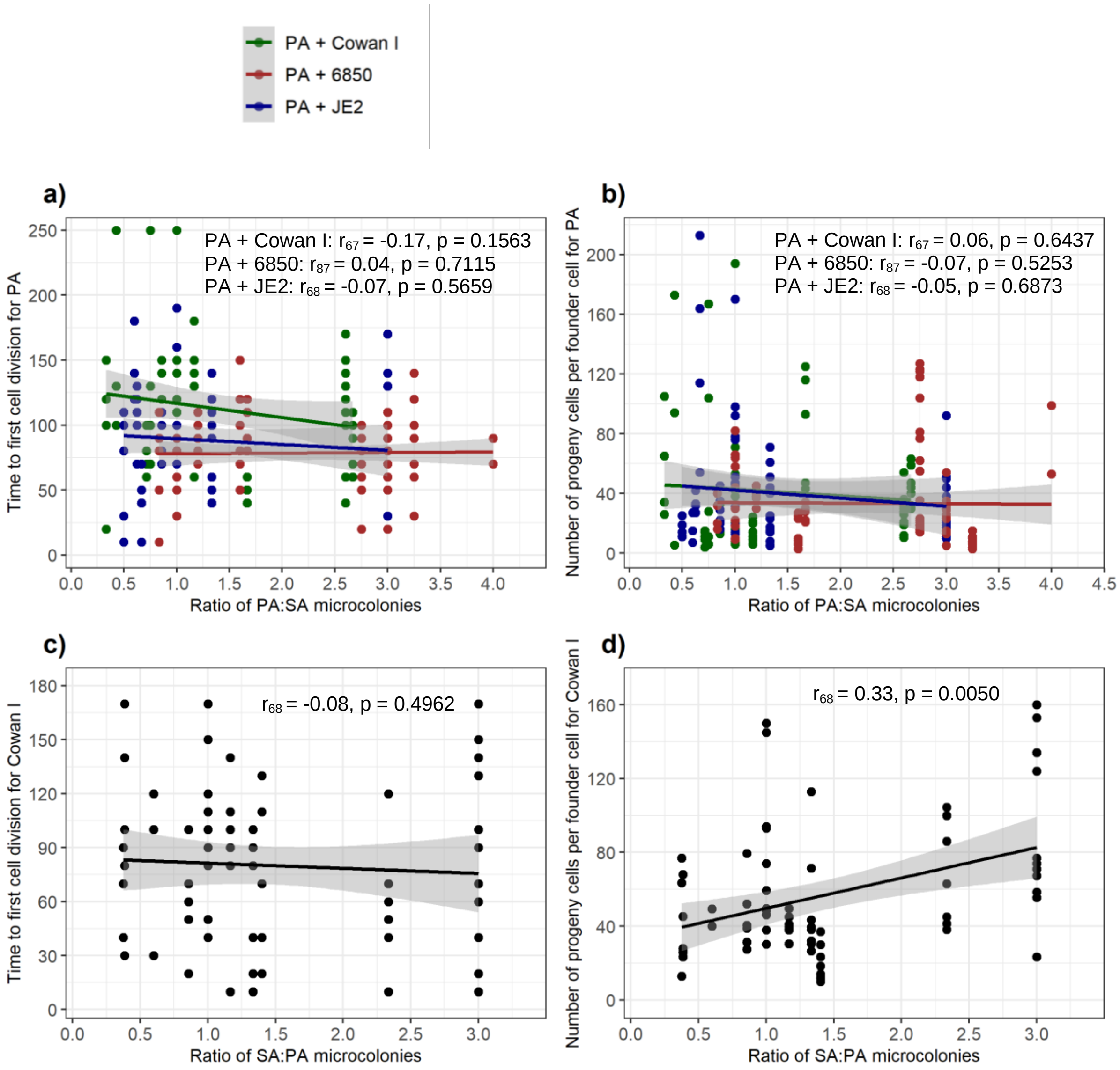

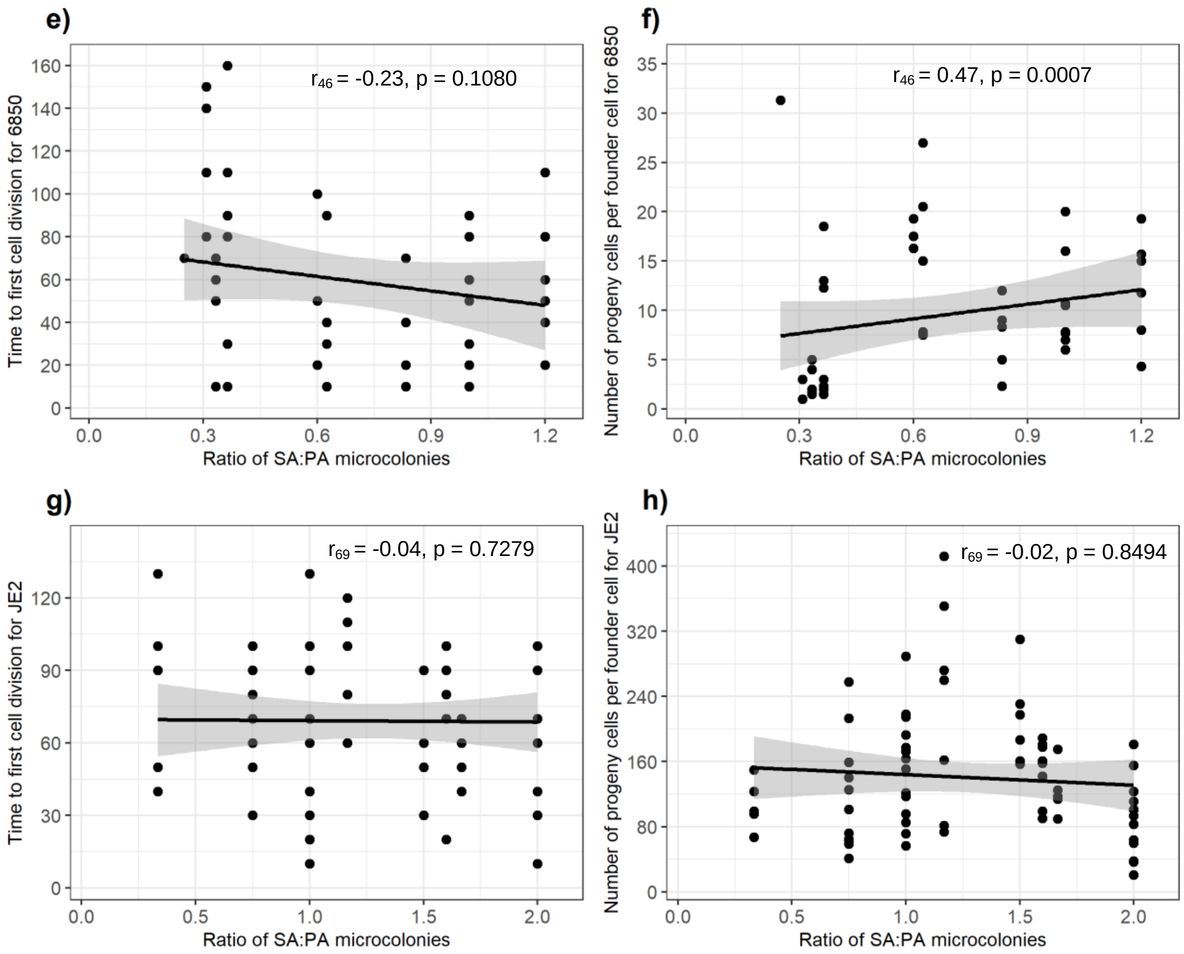

Supplementary Figure S2. Correlation between the time to first cell division (panels a, c, e, g) or the number of progeny cells per founder cell (panels b, d, f, h) and the ratio of founder cells of the two species (PA:SA for PA, panels a + b, and SA:PA for SA strains Cowan I, c + d; 6850, e + f; and JE2, g + h), respectively. With a higher ratio of SA:PA cells, both Cowan I and 6850 produce more progeny cells per founder cell. Note that the x- and y-scales are different in every plot.

Supplementary Figure S3

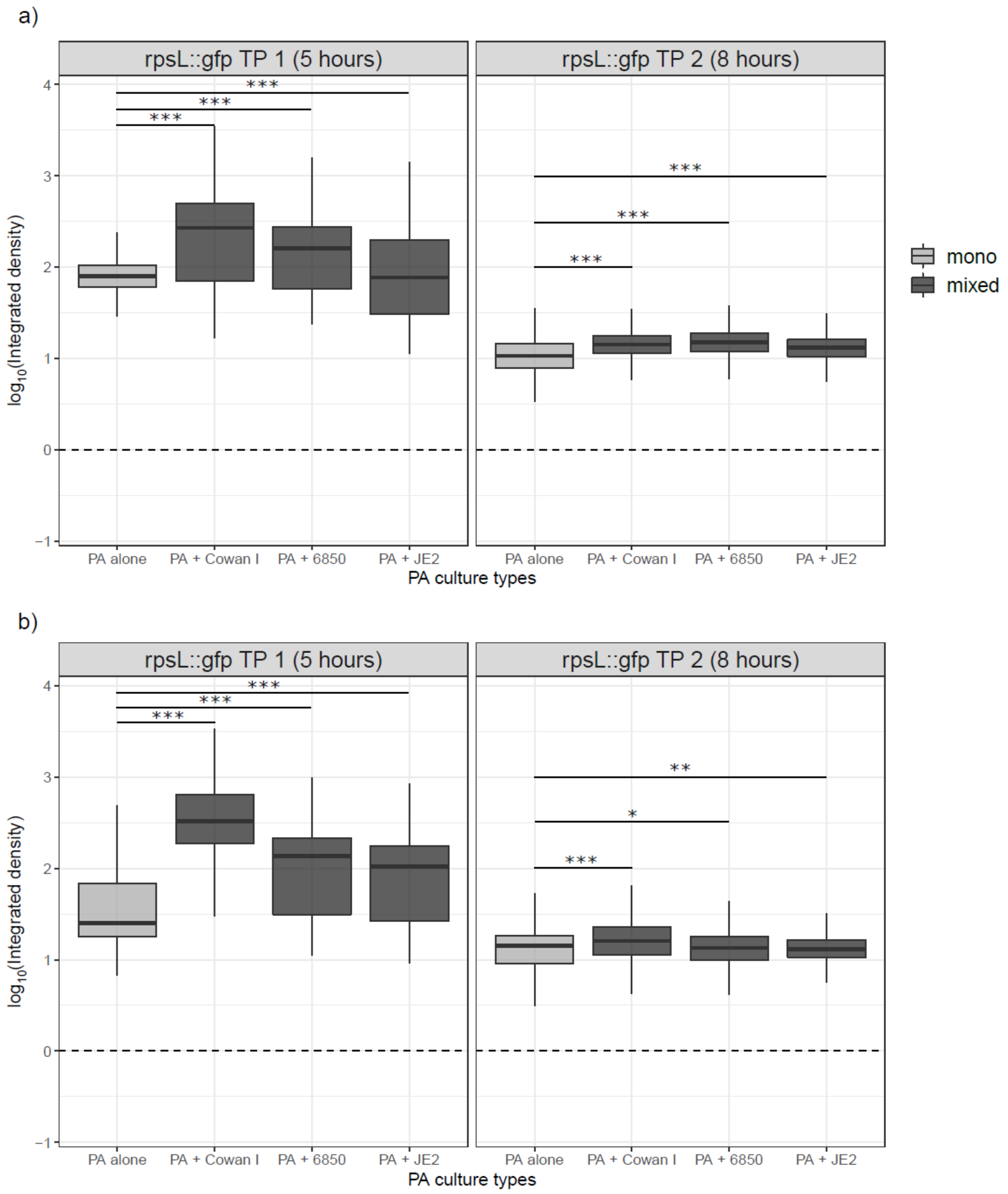

Supplementary Figure S3. Expression of *rpsL::gfp* in the PA *lasR* and *rhlR* double reporters with and without SA, and summary table showing gene expression across treatments and timepoints. We used PA strains harboring transcriptional double reporter strains, where the genes of the quorum sensing regulators *lasR* or *rhlR* are fused to mCherry and the housekeeping gene *rpsL* is fused to GFP (*lasR::mcherry-rpsL::gfp* and *rhlR::mcherry-rpsL::gfp*). We inoculated these strains with (dark-grey)

and without (light-grey) SA strains on agarose patches and took pictures of growing microcolonies at two timepoints, after five hours (TP 1) and eight hours (TP 2) incubation at 37 °C. a) Expression of *rpsL* in the *lasR::mcherry-rpsL::gfp* reporter is more homogeneous than expression of *lasR*. b) Expression of *rpsL* in the *rhlR::mcherry-rpsL::gfp* reporter is more homogeneous than expression of *rhlR*.

Supplementary Figure S4

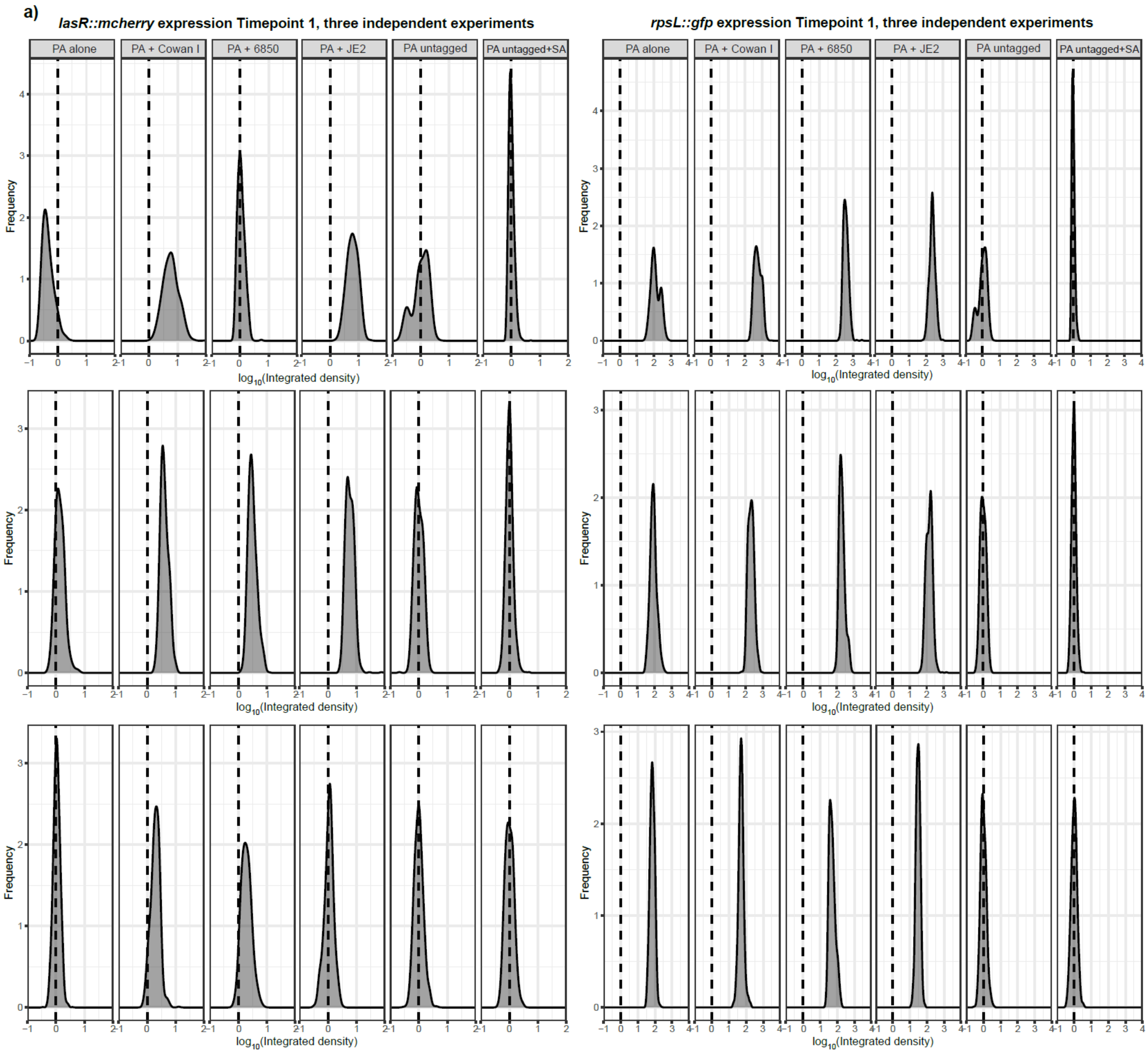

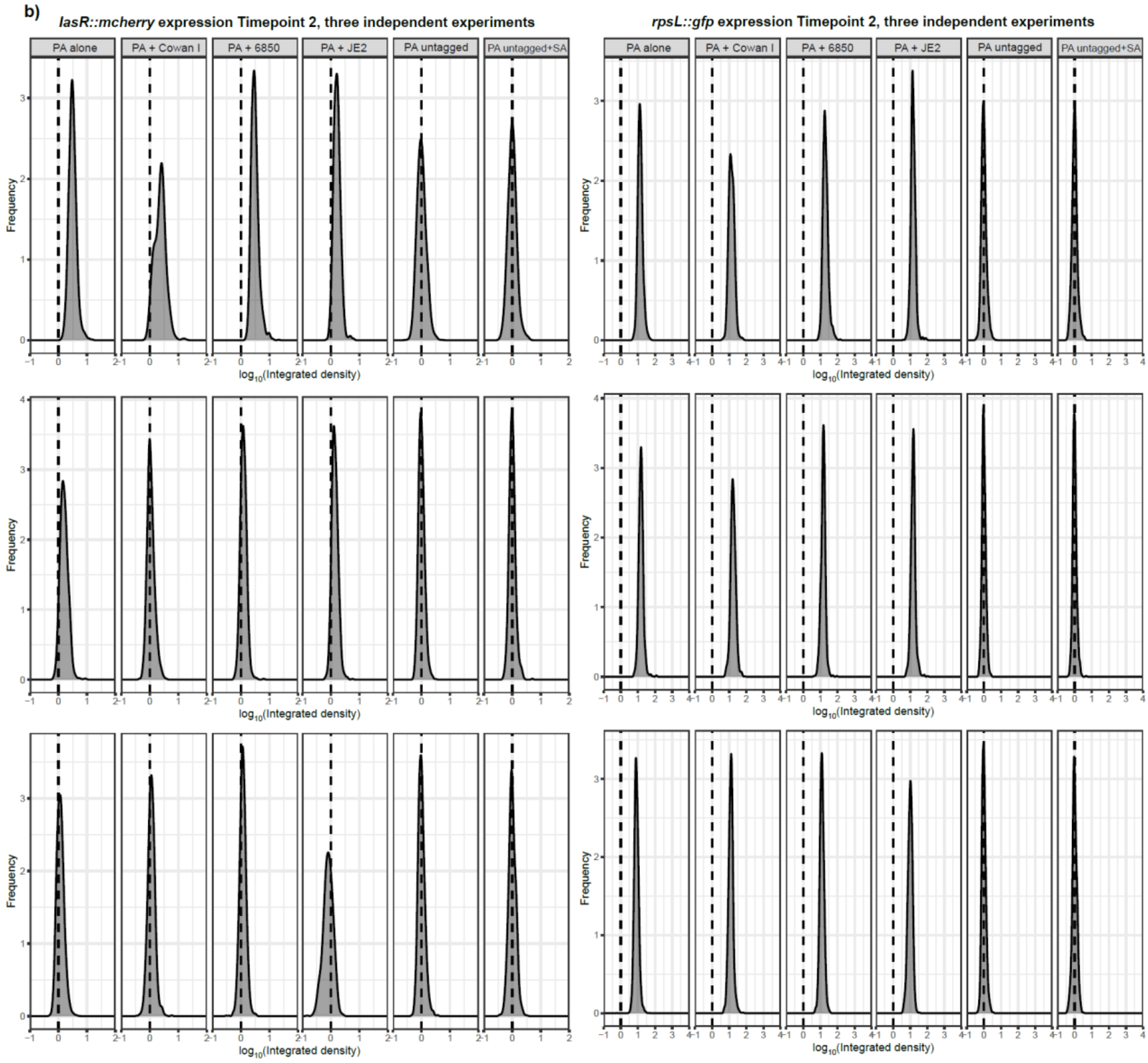

Supplementary Figure S4. Individual histograms for *lasR::mcherry* (left) and *rpsL::gfp* (right) expression in the PA double reporter strain *lasR::mcherry-rpsL::gfp*. a) Timepoint 1 (five hours); b) Timepoint 2 (eight hours). Data is from three independent experiments (each row corresponds to one independent experiment).

Supplementary Figure S5

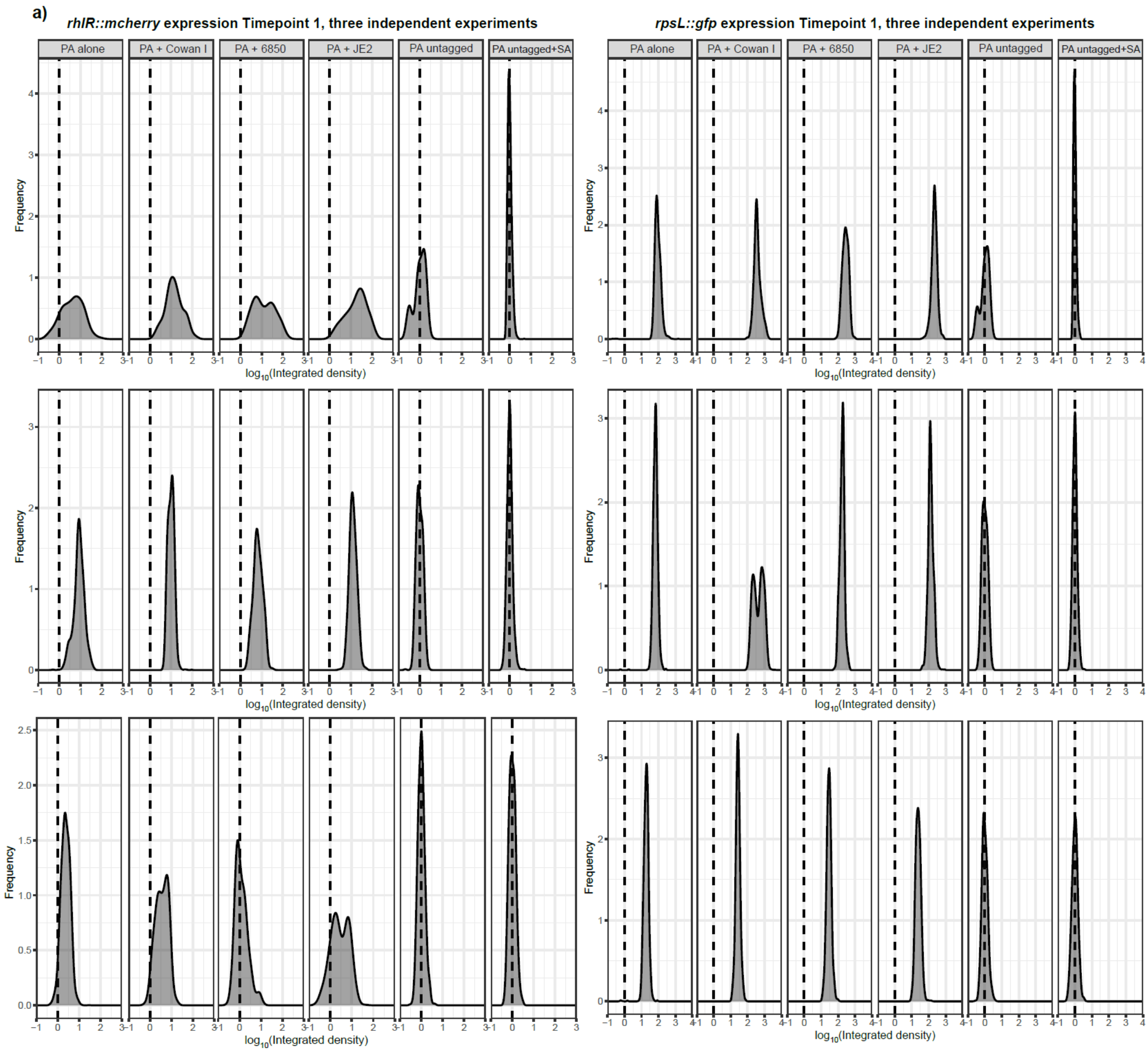

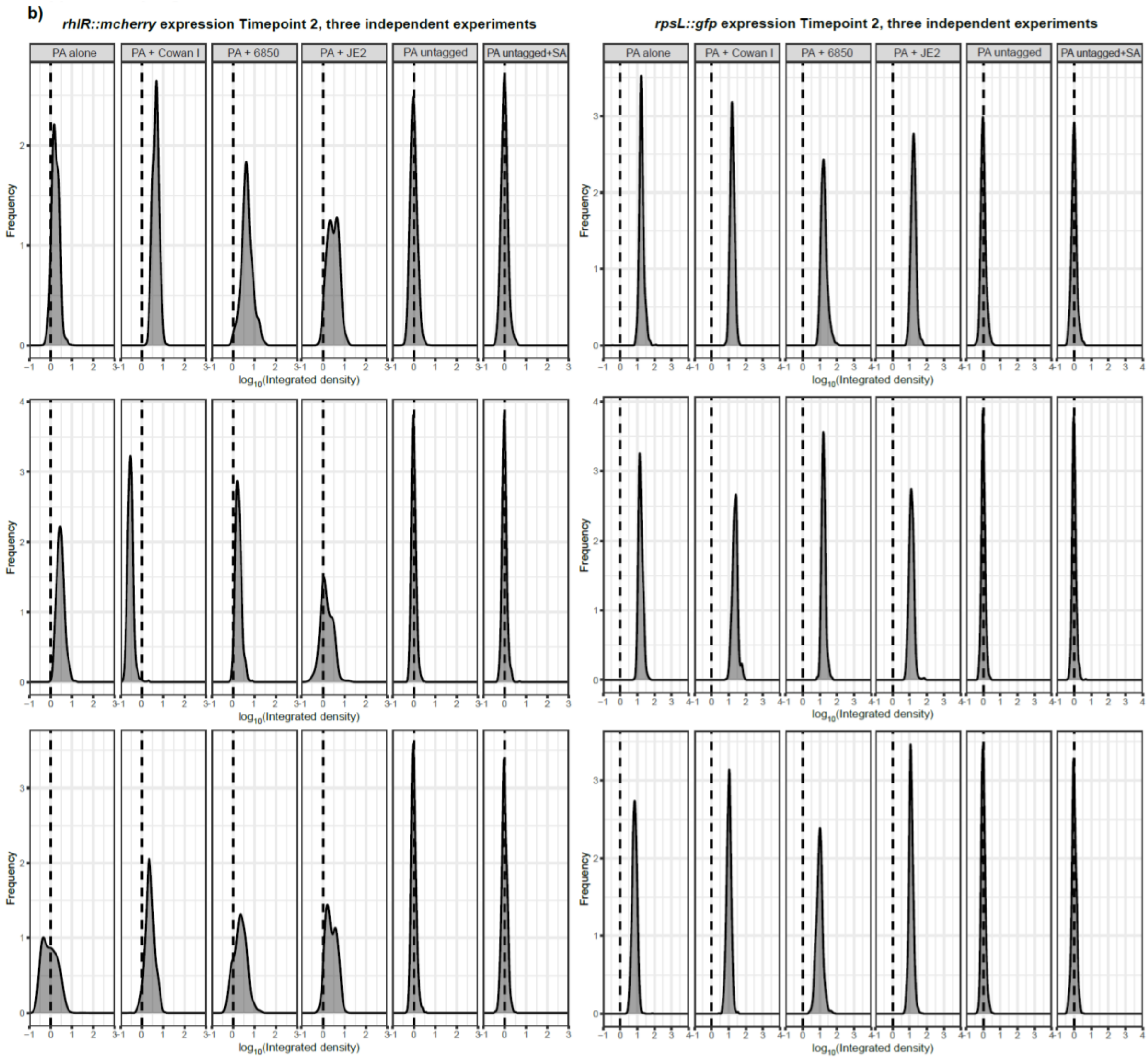

Supplementary Figure S5. Individual histograms for *rhlR::mcherry* (left) and *rpsL::gfp* (right) expression in the PA double reporter strain *rhlR::mcherry-rpsL::gfp*. a) Timepoint 1 (five hours); b) Timepoint 2 (eight hours). Data is from three independent experiments (each row corresponds to one independent experiment).

Supplementary Movie Analysis

All time-lapse movies were acquired over six hours at 37 °C with pictures taken every 10 min. (= timestep). Scale bar at the lower right of each movie represents 10 µm. Only the phase contrast channel is shown. Plots below show the individual growth curves (left-hand side) per microcolony for PA and SA in Movies S1 – S4. Note that some microcolonies are overgrown by other microcolonies during the time-lapse movie and are therefore not well visible anymore at the end of the time-lapse series, which is shown in the snapshot on the right-hand side of each growth curve plot.

Movie S1 analysis. *P. aeruginosa* (PA) growing alone.

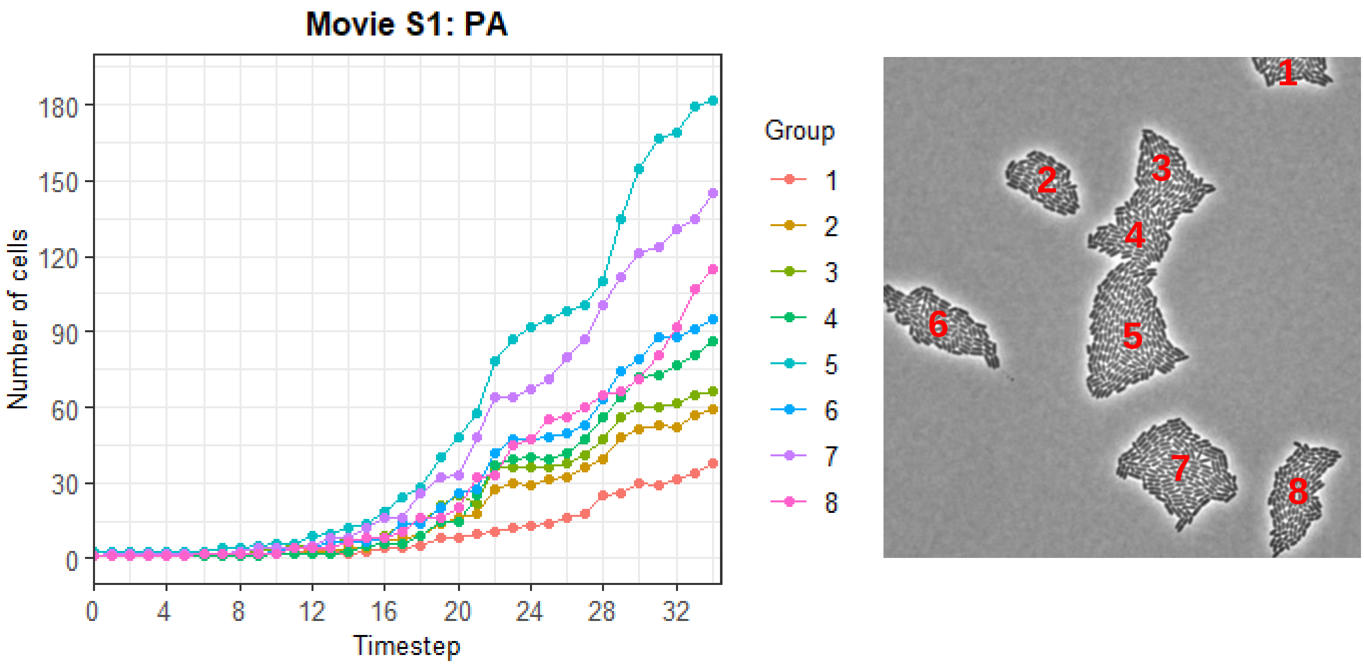

Movie S2 analysis. PA together with the *S. aureus* (SA) strain Cowan I.

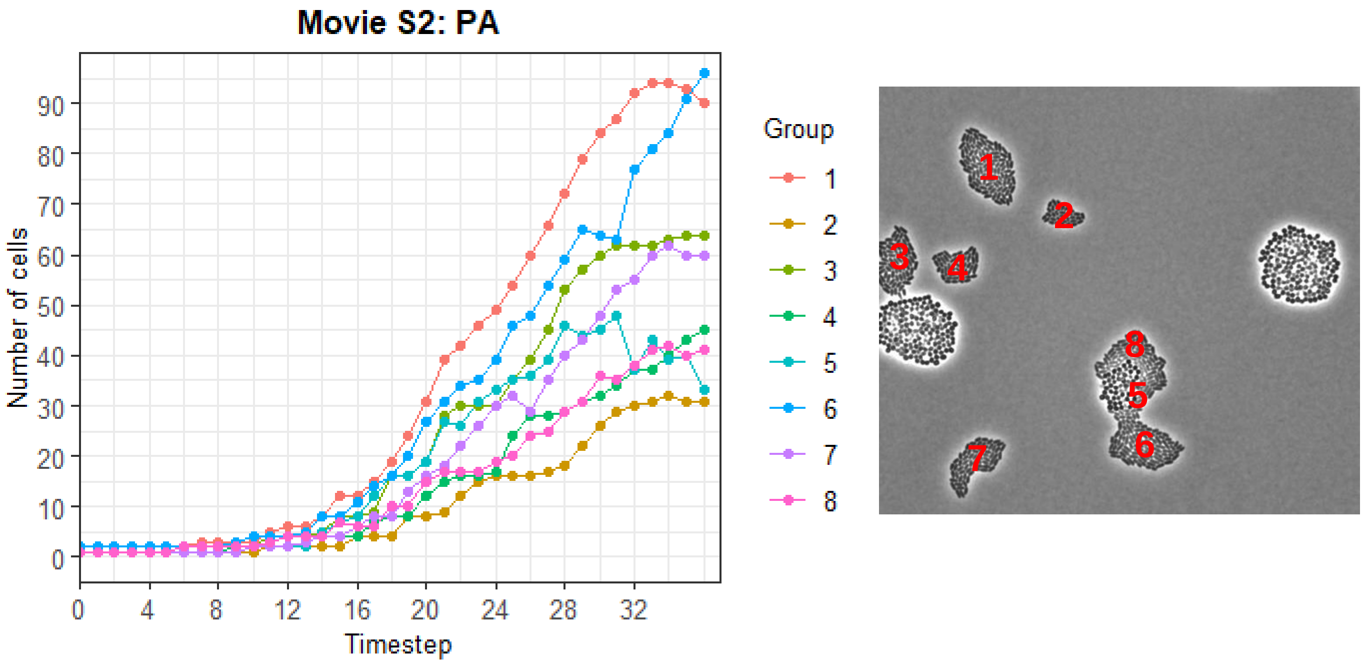

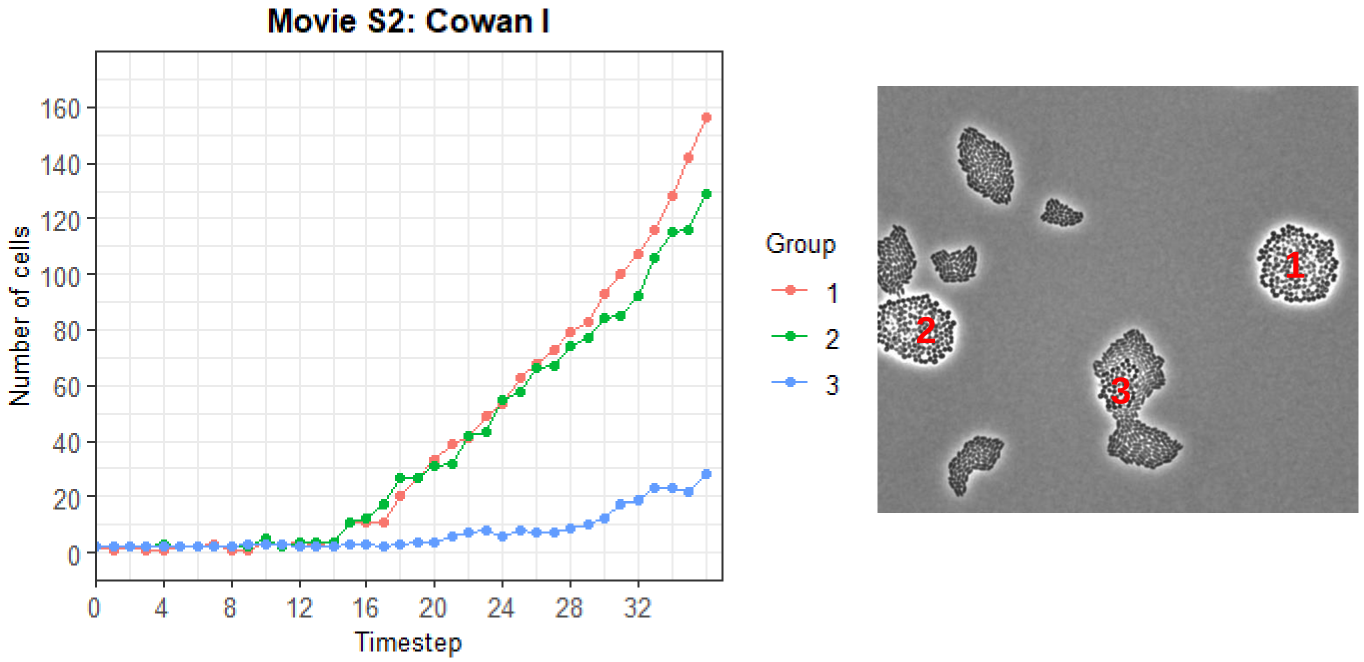

Movie S3 analysis. PA together with the SA strain 6850.

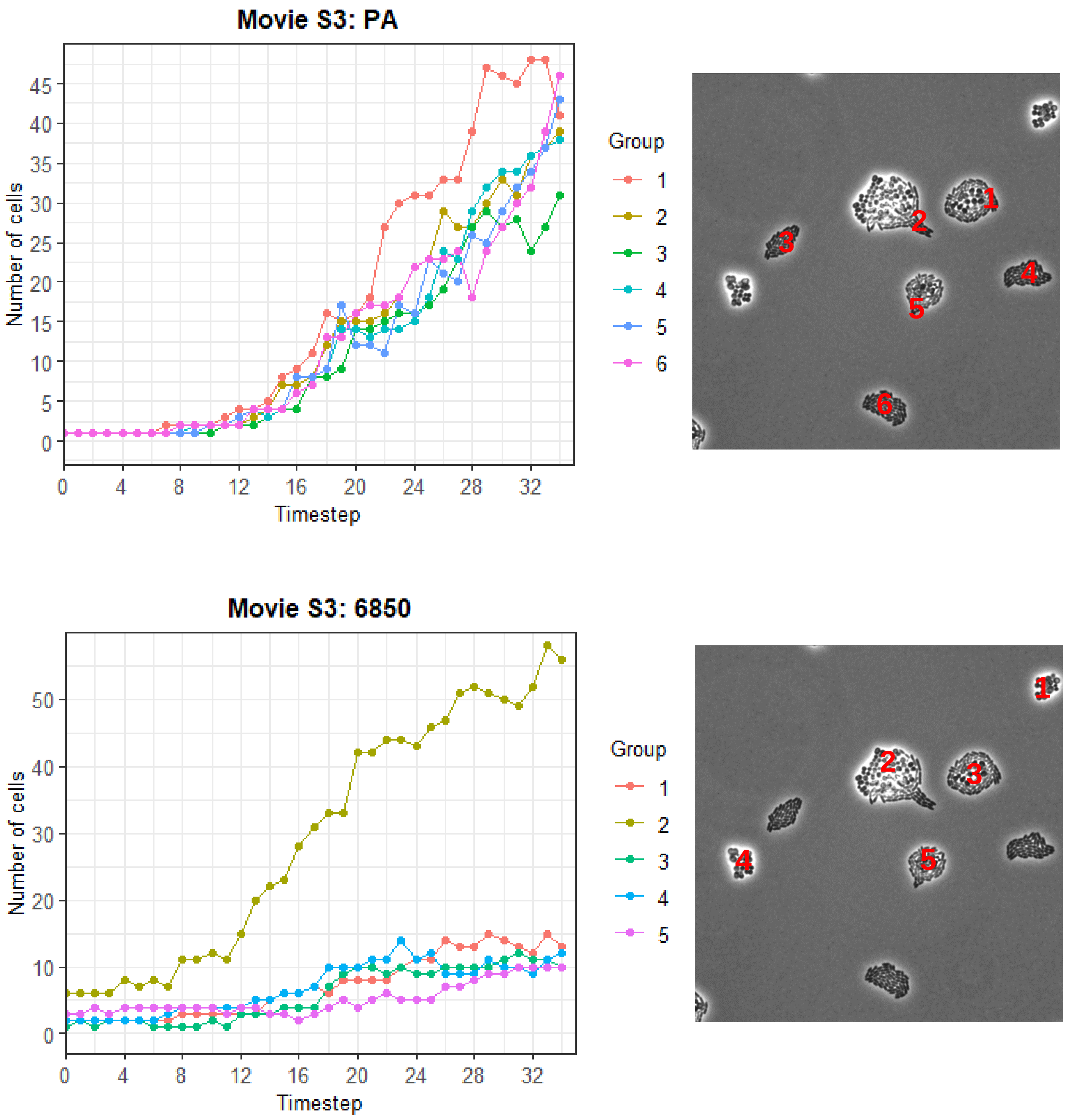

Movie S4 analysis. PA together with the SA strain JE2.

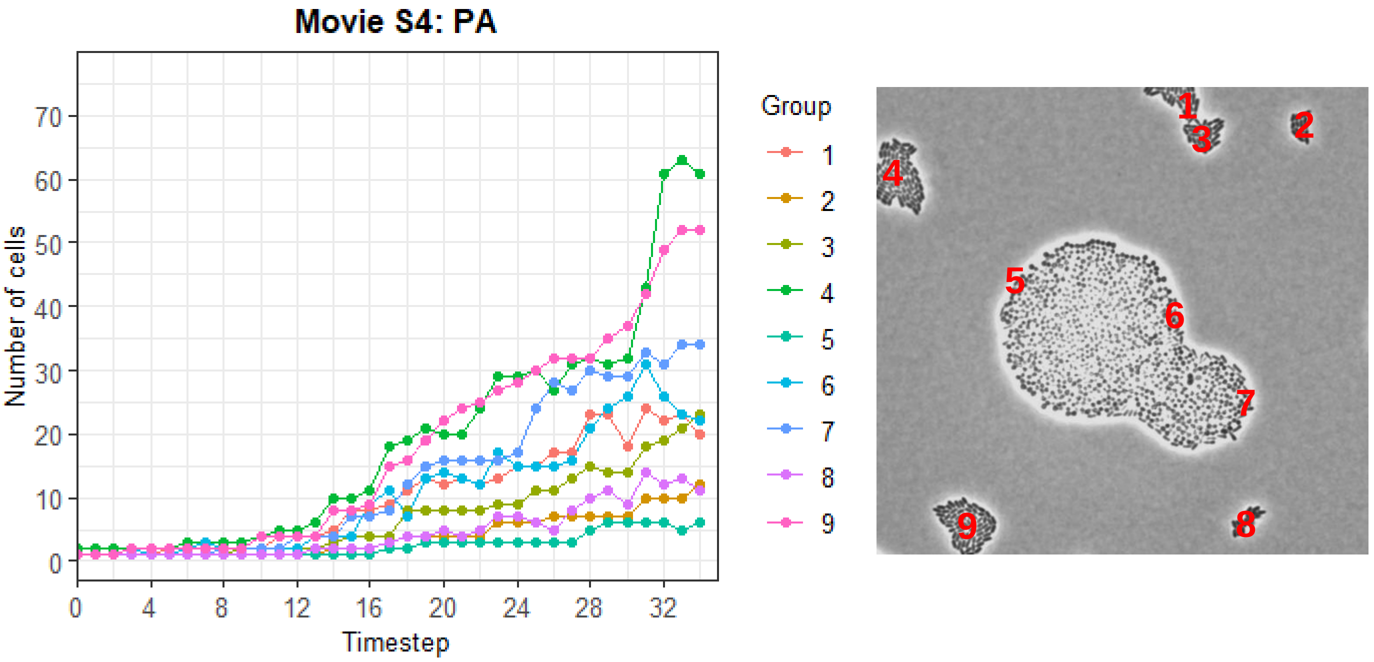

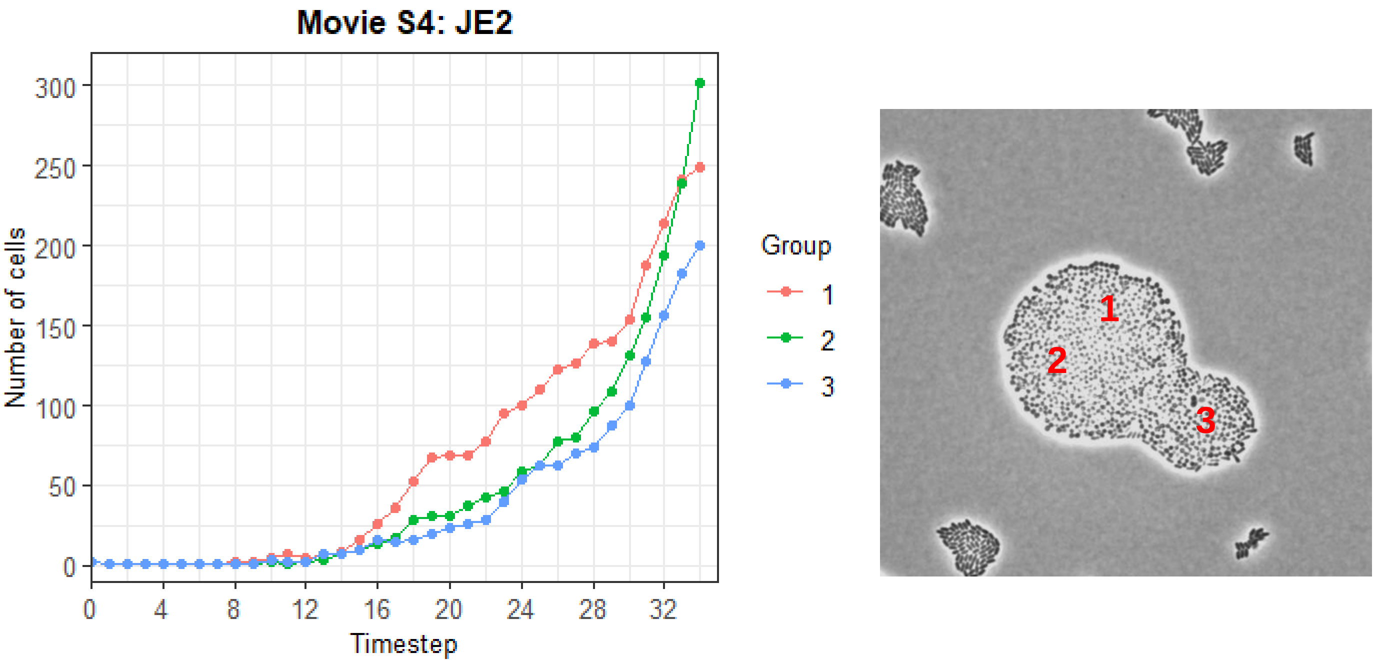

Supplement: Supplementary file 1 [file DataSheet_1.pdf]
